# Chemical Composition and Biological Activities of *Eucalyptus globulus* Essential Oil

**DOI:** 10.3390/plants12051076

**Published:** 2023-02-28

**Authors:** Natália Čmiková, Lucia Galovičová, Marianna Schwarzová, Milena D. Vukic, Nenad L. Vukovic, Przemysław Łukasz Kowalczewski, Ladislav Bakay, Maciej Ireneusz Kluz, Czeslaw Puchalski, Miroslava Kačániová

**Affiliations:** 1 Institute of Horticulture, Faculty of Horticulture and Landscape Engineering, Slovak University of Agriculture, Tr. A. Hlinku 2, 94976 Nitra, Slovakia; 2 Department of Chemistry, Faculty of Science, University of Kragujevac, P.O. Box 12, 34000 Kragujevac, Serbia; 3 Department of Food Technology of Plant Origin, Poznań University of Life Sciences, 31 Wojska Polskiego St., 60-624 Poznań, Poland; 4 Institute of Landscape Architecture, Faculty of Horticulture and Landscape Engineering, Slovak University of Agriculture, Tr. A. Hlinku 2, 94976 Nitra, Slovakia; 5 Department of Bioenergetics and Food Analysis, Institute of Food Technology and Nutrition, University of Rzeszow, Zelwerowicza 4, 35-601 Rzeszow, Poland

**Keywords:** *Eucalyptus globulus*, chemical composition, antimicrobial activity, antioxidant activity, antibiofilm activity, insecticidal activity, vapor phase

## Abstract

*Eucalyptus globulus* essential oil (EGEO) is considered as a potential source of bioactive compounds with significant biological activity. The aim of this study was to analyze the chemical composition of EGEO, in vitro and in situ antimicrobial activity, antibiofilm activity, antioxidant activity, and insecticidal activity. The chemical composition was identified using gas chromatography (GC) and gas chromatography/mass spectrometry (GC/MS). The main components of EGEO were 1,8-cineole (63.1%), *p*-cimene (7.7%), a-pinene (7.3%), and a-limonene (6.9%). Up to 99.2% of monoterpenes were present. The antioxidant potential of essential oil and results indicate that 10 μL of this sample can neutralize 55.44 ± 0.99% of ABTS^•+^, which is equivalent to 3.22 ± 0.01 TEAC. Antimicrobial activity was determined via two methods: disk diffusion and minimum inhibitory concentration. The best antimicrobial activity was shown against *C. albicans* (14.00 ± 1.00 mm) and microscopic fungi (11.00 ± 0.00 mm–12.33 ± 0.58 mm). The minimum inhibitory concentration showed the best results against *C. tropicalis* (MIC 50 2.93 µL/mL, MIC 90 3.17 µL/mL). The antibiofilm activity of EGEO against biofilm-forming *P. flourescens* was also confirmed in this study. The antimicrobial activity in situ, i.e., in the vapor phase, was significantly stronger than in the contact application. Insecticidal activity was also tested and at concentrations of 100%, 50%, and 25%; the EGEO killed 100% of *O. lavaterae* individuals. EGEO was comprehensively investigated in this study and information regarding the biological activities and chemical composition of the essential oil of *Eucalyptus globulus* was expanded.

## 1. Introduction

*Eucalyptus* from the Myrtaceae family is known and used in folk medicine for a number of different ailments and health problems. The plant or essential oil has been used for treatments such as rhinitis, bronchial infection, or to reduce fever [1].

*Eucalyptus* is native to Australia, and it is estimated that there are up to 700 species. It is an evergreen, tall tree that is cultivated for its wood, gum, pulp, oil, as well as for its aesthetic and medicinal value [2]. The essential oil derived from the leaves of this tree is one of the most important of all woody and non-woody trees, due to its wide range of uses [3]. Plant essential oils contain a variety of chemicals and are a mixture of different components such as (such as esters, monoterpenes, aldehydes and ketones, or monoterpenes and sesquiterpenes). Such chemical compositions are involved in the defense mechanisms of plants against pests and microorganisms [3].

The first mention of the use of *Eucalyptus globulus* in Europe was in 1792 in France. Since this moment, the seeds have spread to other European countries and even around the world. Since the 19th century, this species of *Eucalyptus* has been the most widespread throughout the world [4]. *Eucalyptus* are classified as aromatic, medicinal plants and belong to the myrtle family. The essential oil of various *Eucalyptus* species are characterized by antioxidant and antimicrobial properties and is often used in the pharmaceutical industry as well as in the cosmetic industry [5]. The essential oil is also characterized by its preservative ability, since it can prolong the shelf life of foodstuffs, and it is also used as a flavoring agent. Many studies indicate good antimicrobial properties, and it can also inhibit the growth of a wide range of microorganisms [6,7].

The oxygenated and unoxygenated monoterpenes, as well as the oxygenated sesquiterpenes, make up the majority of the essential oil. The most oxygenated monoterpenes among these are eucalyptol (51.62%) and terpinen-4-ol (2.74%), while the main non-oxygenated monoterpenes are α-pinene (23.62%), *p*-cymene (10%), and β-myrcene (8.74%) [5]. The primary ingredients of EGEO are harvested throughout the summer, and one of the most significant elements impacting the quantity of medicinal plant efficacy is the period of plant organ harvesting. Traditional medicine makes extensive use of *Eucalyptus*, and its essential oil is one of the most popular and effective medications. Many of these effects are most likely caused by 1,8-cineole, which is the active component. The oxygenated monoterpene composition of 1,8-cineole is the primary element shared by all *Eucalyptus* species, and the main difference is simply in the quantity of this compound, according to a comparison of the active components of *Eucalyptus* species essential oil [8]. The chemical composition of the leaf essential oils of *Eucalyptus globulus* Labill plants growing at 1347 and 3191 m elevation in the Caar canton of Ecuador was the same, with 1,8-cineole accounting for more than 67% of the total composition. There were no discernible variations between the two chemical tends to make of the essential oils [9].

*Eucalyptus* species and their essential oils are widely used, and the United States Food and Drug Administration as well as the Council of Europe have evaluated their use as safe and non-toxic [3].

Antibiotic resistance is increasing rapidly, and other options need to be explored, such as natural products, including EGEO, which may have an inhibitory effect on microorganisms in vitro and in situ vapor phases, with an antibiofilm effect, possibly as a bioinsecticide.

This study’s major objectives were to identify the chemical components of *Eucalyptus globulus* essential oil (EGEO) using the GC–MS method, as well as EGEO’s antibacterial activity in vitro and in situ as well as its antibiofilm, antioxidant, and insecticidal properties. Our aim was to analyze the EGEO because it has not been studied in such a broad context so far.

## 2. Results

### 2.1. Chemical Composition of Eucalyptus Globulus Essential Oil

Results presented in Table 1 and Table 2 show the volatile composition of EGEO. Identified compounds and their percentage of the amount in the examined oil sample are shown in Table 1, while Table 2 shows the volatiles presented in a percentage for each class of compounds. Overall, in the sample of EGEO identified, there was a total of 99.7% represented by 27 compounds. Obtained results indicate that 1,8-cineole, belonging to the class of monoterpene epoxides, represents the major compound in this sample. Accordingly, monoterpene epoxides are identified as the major class of compounds in this sample, with the total amount of 63.1%. A second class of compounds with a high percentage abundance were monoterpene hydrocarbons. Among them, the dominant were *p*-cimene (7.7%), α-pinene (7.3%), α-limonene (6.9%), γ-terpinene (3.6%), β-pinene (3.0%), and β-myrcene (1.7%). Other identified volatiles were presented in amounts less than 1.5%.

### 2.2. Antioxidant Activity of Eucalyptus Globulus Essential Oil

The antioxidant potential of essential oil obtained from the *E. globulus* was determined by the means of its capacity to neutralize a stable ABTS radical cation. As a positive control in this experiment, the standard compound Trolox was used. The obtained results indicate that 10 μL of this sample can neutralize 55.44 ± 0.99% of ABTS^•+^, which is equivalent to 3.22 ± 0.01 TEAC. Considering that the IC50 value of Trolox is 2.96 ± 0.01 mg/L, tested essential oil shows very good antioxidant potency.

### 2.3. Antimicrobial Activity of Eucalyptus Globulus Essential Oil

The antimicrobial activity of the EGEO, to inhibit the growth of gram-positive (G^+^) and gram-negative bacteria (G^−^), yeasts, and fungi, was determined via two methods. One of the methods is the disc diffusion method and the other is using a minimal inhibitory concentration (MIC 50 and MIC 90), a concentration of essential oil that can inhibit the growth of microorganisms by 50% and 90%.

EGEO (Table 3) achieved the highest efficiency against the yeast *C. albicans* via the disc diffusion method, with an inhibition zone radius of 14.00 ± 1.00, and for *C. albicans*, the essential oil achieved an antimicrobial effect, which we define as a strong activity. Strong activity is also recorded against all tested fungi *A. flavus* (11.00 ± 0.00 mm), *B. cinerae* (11.67 ± 0.58 mm), and *P. citrinum* (12.33 ± 0.58 mm). EGEO showed moderate activity against both G^+^ bacteria (*B. subtilis*, *S. aureus*), G^−^ bacteria (*S. enterica*, *Y. enterocolitica*), and yeasts (*C. glabrata*, *C. tropicalis*), with inhibition zone sizes ranging from 5–10 mm. Weak activity was shown against G^−^ biofilm-forming bacteria (*P. fluorescens* biofilm), *P. aeruginosa*, and the yeast *C. krusei*, and the weakest antimicrobial effect was exhibited by the EGEO against the G^+^ bacteria *E. faecalis*, with the inhibition zones ranging from 2.33 ± 0.58 mm to 4.33 ± 0.58 mm.

Table 4 also shows the results of the second antimicrobial method, the minimal inhibitory concentration, or the concentration of essential oil that can inhibit the growth of microorganisms by 50% and 90%. The lower the concentration of EGEO, the more potent activity the essential oil showed. The strongest activity, namely MIC 50 2.93 µL/mL (MIC 90 3.17 µL/mL), was confirmed against the yeast *C. tropicalis* and against the yeast *C. krusei* (MIC 50 5.86 µL/mL, MIC 90 6.31 µL/mL). The highest concentration of essential oil and the weakest activity was exhibited by the essential oil against G^−^ *P. aeruginosa* (MIC 50 374.02 µL/mL, MIC 90 397.64 µL/mL) and against G^+^ *B. subtilis* (MIC 50 374.02 µL/mL, MIC 90 397.64 µL/mL).

In this work, we also tested the effect of different concentrations on antifungal activity against selected species of microscopic fungi (Table 5). Compared to the results in Table 3, where a 100% concentration of EGEO was tested, Table 5 shows the activity of essential oil against microscopic fungi at concentrations of 500, 250, 125, and 62.5 µL/mL diluted in DMSO. EGEO was most effective against *A. flavus* at a concentration of 500 µL/mL (inhibition zone 7.67 ± 0.58 mm) and least effective at a concentration of 62.5 µL/mL (inhibition zone 3.33 ± 1.53 mm). The inhibition zone size was 7.33 ± 2.89 mm, the strongest antifungal activity of EGEO was produced at a concentration of 125 µL/mL against *B. cinerea*, and the smallest inhibition zone was produced at a concentration of 250 µL/mL (inhibition zone 4.33 ± 2.06 mm). Only a concentration of 500 µL/mL was effective against *P. citrinum* (inhibition zone 4.33 ± 1.53 mm); the other concentrations had no effect on growth inhibition.

### 2.4. Antimicrobial Analysis In Situ

The antimicrobial activity of EGEO in the vapor phase was tested against different microorganisms and at different concentrations in this work (Table 6).

The antimicrobial activity of EGEO in the vapor phase was tested against different microorganisms and at different concentrations in this work (Table 6). The concentrations of essential oil used were 500 µL/L, 250 µL/L, 125 µL/L, and 62.5 µL/L, and the food model for testing the antimicrobial activity against G^+^/G^−^ bacteria, yeasts, and microscopic fungi was white radish. The most effective concentration against the selected G^+^ bacteria was 62.5 µL/L, namely against *E. faecalis* (76.86 ± 1.43%) and *S. aureus* (76.79 ± 2.15%). All other concentrations had an inhibitory effect against G^+^ bacteria, although a weaker one, however; EGEO in the vapor phase showed probacterial activity against *B. subtilis*, thus promoting growth at concentrations of 500 µL/L, 125 µL/L, and 62.5 µL/L. Against G^−^ bacteria, the essential oil exhibited up to 91.26 ± 4.58% against *Y. enterocolitica* in the vapor phase at a concentration of 500 µL/L. Vapor phase essential oil had the weakest effect against *P. aeroginosa* at a 62.5 µL/L concentration of 8.62 ± 1.06%. On the biofilm-producing bacteria *P. flourescens*, the biofilm had a probacterial activity (−54.41 ± 2.13%) at a concentration of 250 µL/L. The most effective concentration against yeast was 62.5 µL/L, against *C. glabrata* (86.82 ± 3.07%), and at the same time this concentration was the least effective against *C. albicans* (5.97 ± 1.36%). EGEO had pre-fermentation activity in the vapor phase against *C. glabrata* (125 µL/L) and *C. tropicalis* (500 µL/L). In this work, three microscopic fungi and the antimicrobial effect of EGEO in the vapor phase on white radish were also tested. All concentrations had an inhibitory effect on mycelial growth in all microscopic fungi. The most effective concentration was 62.5 µL/L against *P. citrinum* with an inhibitory effect of 85.77 ± 1.7%. The least effective concentration was 500 µL/L against *B. cinerea*, with 11.70 ± 0.87% activity.

### 2.5. Analysis of Biofilm Developmental Phases and Evaluation of Molecular Differences on Different Surfaces Using MALDI-TOF MS Biotyper

The antibiofilm effect of EGEO on anticorrosive and plastic surfaces against *P. fluorescens* was evaluated using MALDI-TOF MS Biotyper mass spectrometry. The spectra of the control and planktonic control samples developed identically; therefore, the planktonic spectra were used as representative spectra of the control group. They were compared with the experimental groups with the addition of EGEO on the anticorrosion and plastic surfaces.

**Figure 1:**
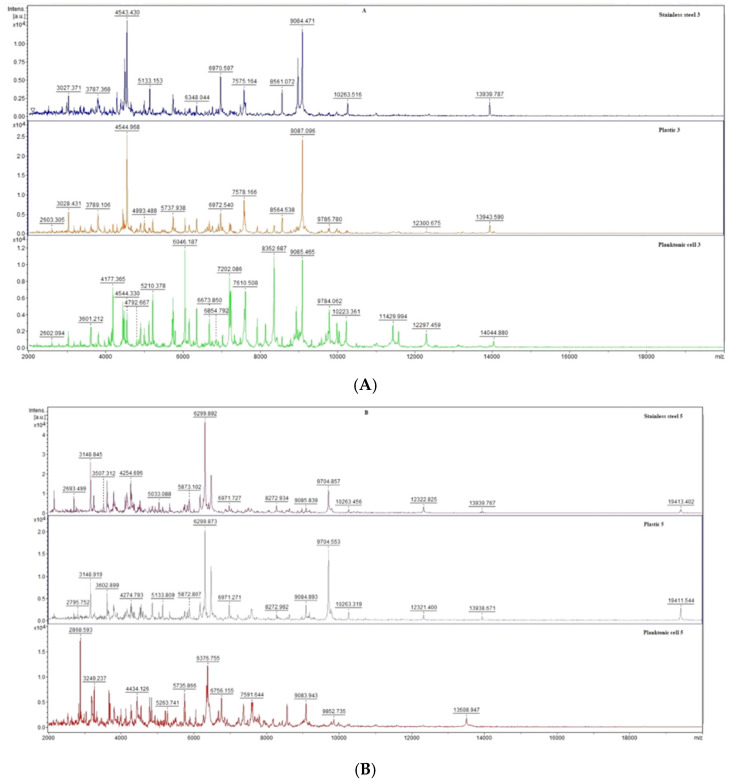

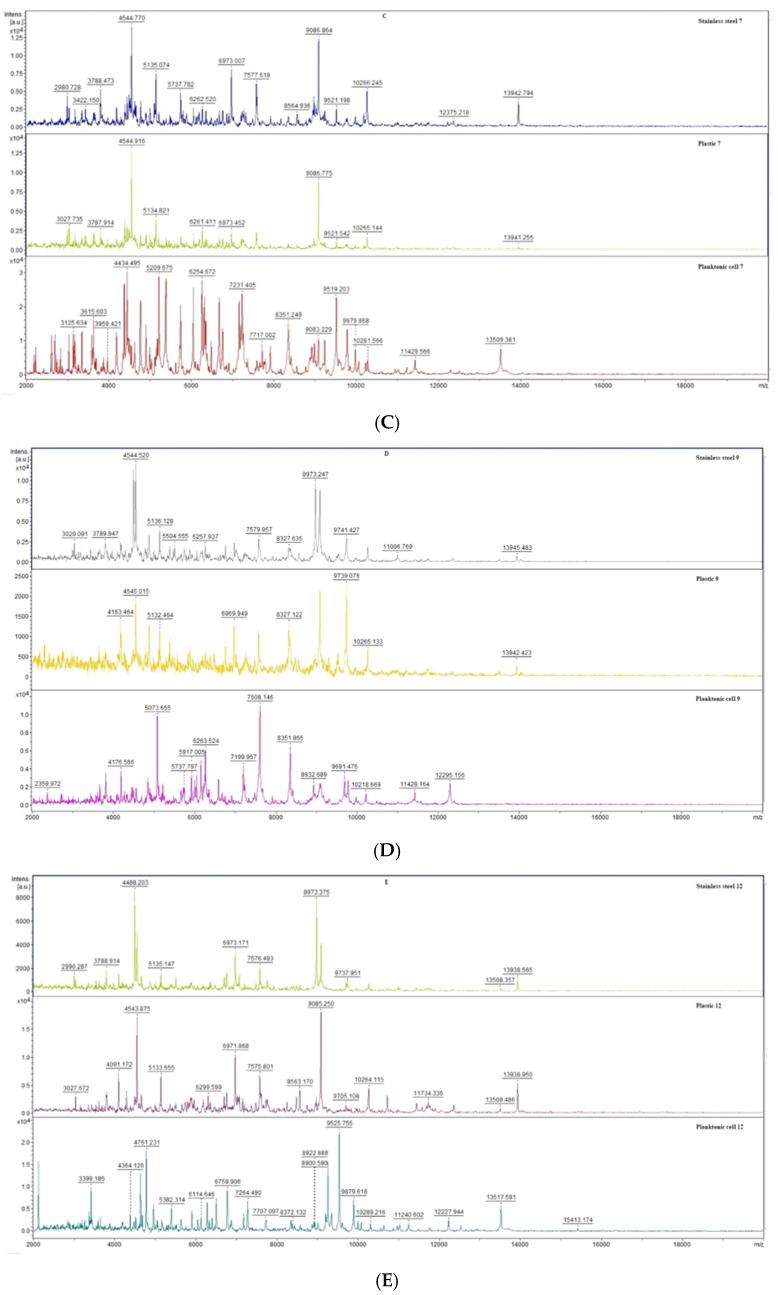

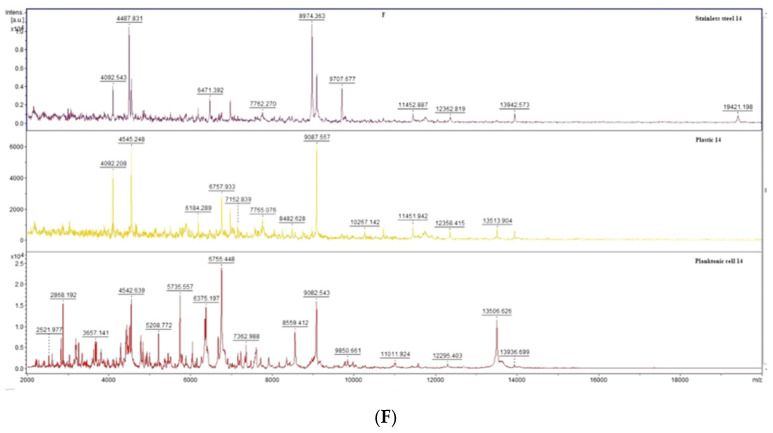
MALDI-TOF mass spectra of *P. fluorescens* biofilm development after EGEO exposition: (**A**)—3rd day; (**B**)—5th day; (**C**)—7th day; (**D**)—9th day; (**E**)—12th day; (**F**)—14th day.

Already from the beginning of the experiment on day 3 of the analysis (Figure 1A), differences in the experimental group compared to the control planktonic spectrum were observed in the number of peaks as well as the shape of the mass spectrum. A lower number of peaks was detected for the spectrum obtained from the anticorrosive surface compared to the experimental spectrum on the plastic surface. On day 5 of the experiment (Figure 1B), the differences in the experimental spectra compared to the control planktonic spectrum was also noted, while the similarity of the experimental spectra in both the peak shape and number of peaks was observed. The trend of dissimilarity between the control and experimental groups was maintained throughout the experiment (Figure 1C–F). Differences were observed between the experimental groups, while the degradation and inhibition of the *P. fluorescens* biofilm via the application of EGEO was observed in both groups, which is evidence that this essential oil is able to disrupt the biofilm homeostasis in the early stages and is thus a suitable alternative to combat this biofilm.

**Figure 2:**
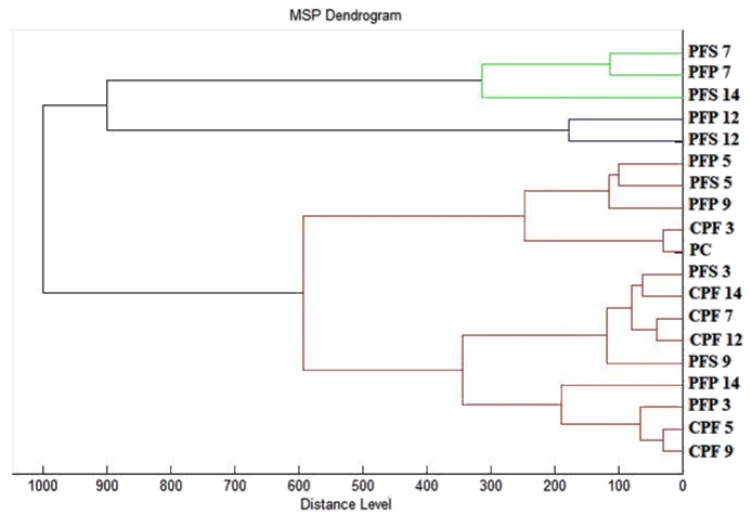
Dendrogram of *P. fluorescens* biofilm progress after EGEO exposition. PF—*P. fluorescens*; C—control; S—stainless steel; P—plastic; P—planktonic cells.

To visualize the similarity of the mass spectra, a dendrogram was constructed based on the MSP distances, and we can observe how the similarity evolved from day to day (Figure 2). The constructed dendrogram shows that the biofilms of the experimental group had the highest similarity in the early stages during day 3 (PFS 3 and PFP 3) along with the planktonic cells (PC) and controls (CPF 3−14). There was an increase in the MSP distances of the experimental groups over the days, with the greatest distance observed on day 14 of the experiment (PFS 14 and PFP 14), with a dominance on the anticortical surface (PFFS 14). The observations of the evolution of MSP distances based on the constructed dendrogram are in agreement with the analysis of the mass spectra and tell about the inhibitory effect on *P. fluorescens* biofilms produced by the influence of EGEO.

### 2.6. Insecticidal Activity of Eucalyptus Globulus Essential Oil

The insecticidal activity of EGEO was tested on 30 individuals of *Oxycarenus lavaterae* (Table 7). The concentrations of essential oil tested were 100%, 50%, 25%, 12.5%, 6.25%, and 3.125%. When the activity was 100%, EGEO killed 30 individuals out of 30. The concentration of 12.5% showed the number of individuals killed was eighteen, hence the insecticidal activity was 60%; when the concentration killed six individuals, the insecticidal activity was 20%. The weakest insecticidal efficacy was found for the 3.125% essential oil concentration, where only two individuals were killed, and the activity was 6.66%. The results suggest that as the concentration decreases, the insecticidal activity also decreases.

## 3. Discussion

Several previous studies show that *Eucalyptus* species belong to the 1,8-cineole chemotype, regardless of the cultivation place [5,6,10,11,12,13,14]. The only difference between these results is in the amount of identified 1,8-cineole, which ranges from 17.2% to 90.0% of the total [12]. Bearing that in mind, our study is in agreement with previously published data. This monoterpene epoxide has been widely studied for its pharmacological properties, including antimicrobial, anti-inflammatory, and antioxidant properties [12,14,15,16]. Moreover, it is used as a flavoring agent in many cosmetic products, or as a fragrance in products such as bath additives, mouthwashes, and insect repellants [17]. Besides this monoterpene epoxide, the sample tested in this study is characterized with notable amounts of monoterpene hydrocarbons, *p*-cimene (7.7%), α-pinene (7.3%), α-limonene (6.9%), γ-terpinene (3.6%), β-pinene (3.0%), and β-myrcene (1.7%). Previous reports show that EGEOs are also characterized by higher amounts of sesquiterpenes such as aromadendrene, spathulenol, globulol, and [18,19,20]. In the sample examined in this study, out of the sesquiterpenes identified, only a small amount, 0.4%, was aromadendrene. Observed differences in the EO chemical composition of the same plant species can be a result of genetic differences and variations in environmental factors, such as harvesting seasons, geographical location, climate, etc. [18]. Previous studies have confirmed that minor constituents play a role in the antibacterial activity, probably by creating synergistic effects with other constituents present in EGEO [21]. Various studies also suggest the synergistic effects of combinations such as limonene/1,8-cineole, cinnamaldehyde/eugenol, thymol/eugenol, carvacrol/eugenol, and thymol/carvacrol [16,22,23].

Essential oils have been generally identified as good antioxidants. This feature is associated with Eos, mainly owing to their capability to reduce free radical formation and to scavenge free radicals. In food science, the use of essential oils as antioxidants is a field of growing interest. Regardless, their antioxidant potency is in close relationship with their chemical composition [5,6,10,11,12,13,14,15,16,17,18,19,20,24,25]. Generally, compounds in high abundance, such as thymol, carvacrol, linalool, 1,8-cineole, geranial/neral, citronellal, isomenthone, and menthone, play a key role in their antioxidant properties [25]. Our results indicate that 10 μL of this EO can neutralize 55.44% of the ABTS radical cation, which can be explained by the relatively high abundance of 1,8−cineole. EGEOs exhibited very strong anti-radical activity against the DPPH radical [26].

In our study, the antimicrobial effect against 14 microorganisms was investigated. The antimicrobial activity of EGEO against fish pathogenic bacteria was also researched in a study by Park et al. [27]. The inhibitory activity of the essential oil was tested via the disc diffusion method. According to the disc diffusion test, as the concentration of EGEO increases, the size of the inhibition zone and hence the activity increases. These results show that EGEO has antimicrobial activity against all the seven bacteria tested in their work, and there was no significant difference found among the genera. Our results via the disk diffusion method indicate that EGEO is effective on both G^+^ and G^−^ bacteria, as well as yeasts and microscopic fungi. Antimicrobial activity ranges from a weak to moderate to strong activity, especially against microscopic fungi and the yeast *C. albicans*. Boulekbache-Makhlouf et al. [28] tested the crude extract of *Eucalyptus globulus* fruits for antibacterial properties against *S. aureus*, *B. subtilis*, and *K. pneumoniae* via the disc diffusion method. The extract produced zones of inhibition ranging in size from 5.5 ± 0.5 mm (*B. subtilis*) to 8.67 ± 0.58 mm (*S. aureus*). Our results showed stronger antimicrobial activity and larger inhibition zones against *B. subtilis* (6.67 ± 0.58 mm) than against *S. aureus* (5.67 ± 0.58 mm). This may be due to the different chemical composition of *Eucalyptus globulus* extract and essential oil. The results obtained showed that EGEO has antimicrobial activity against G^−^ bacteria (*E. coli*) as well as G^+^ bacteria (*S. aureus*) [29]. The antimicrobial effect of the essential oil was evaluated and showed a strong inhibitory effect on 17 food spoilage microorganisms. The diameter of the inhibition zone ranged from 15 to 85 mm for G^+^ bacteria and from 10–49 mm for yeasts [30]. The antimicrobial activity of EGEO has been confirmed in several studies, as confirmed by our results [31,32,33,34,35].

EGEO also showed strong antibacterial activity in the work of Boulekbache-Makhlouf et al. [28] against *B. subtilis* and *S. aureus*. The minimal inhibitory concentration (MIC) values measured 30 μg/mL and 80 μg/mL. These results suggest that *E. globulus* fruits have an interesting antibacterial activity. The minimal inhibitory concentration (MIC) against *B. subtilis* and against *S. aureus* in our study ranged from MIC 50 140.25–374.02 μL/mL (MIC 90 334.72–397.64 μL/mL). The differences in the results may be on the basis that in our study we used essential oil from the whole plant, and in the work of Boulekbache-Makhlouf et al. (2013) they tested crude extract from the fruit of *Eucalyptus globulus*. In this study by Mekonnen et al. [36], the in vitro antimicrobial activities of *E. globulus* against *Trichophyton* and *Aspergillus* were demonstrated. The minimal inhibitory concentration values were in the range of 11 ± 1.3–27.3 ± 1.10 mg/mL. The essential oil was extracted from fresh leaves, which were harvested and subsequently prepared into essential oil, which also confirms that essential oils have antimicrobial efficacy not only against bacteria but also microscopic filamentous fungi. The essential oil of leaves and the bark of *E. globulus* showed inhibitory activity against *B. subtilis* (MIC = 100; 6250 μg/mL) [37,38]. Results similar to ours demonstrate that the EGEO has antimicrobial efficacy against several bacteria. Antimicrobial activity was also tested by [10] using the minimal inhibitory concentration (MIC) against the bacteria and yeasts. The MIC ranged from 2.25 to 9 mg/mL for the bacteria and from 1.13 to 2.25 mg/mL for the yeast. Significantly better antimicrobial activity was demonstrated in the vapor phase of the essential oil.

Kačániová et al. (2017) tested on apple, carrot, and potato how the essential oil of *Salvia officinalis* can inhibit the growth of *B. subtilis* [39]. The most effective inhibition was 78.45% at 125 μL/L on apple and the lowest was 1.89% at 125 μL/L on carrot. EGEO at concentrations of 62.5, 125, and 500 µL/L had probacterial efficacy, as well as at a 250 µL/L concentration (24.92 ± 1.68%). Studies claim that there is still no standard methodology for assessing the antimicrobial activity of vapor phase essential oils, but studies conducted so far suggest that vapor phase essential oils could be used in food preservation [40]. Our results confirm that EGEO has antimicrobial properties in the vapor phase.

In the study by Goldbeck et al. (2014), the aim was to evaluate whether essential oils from *Eucalyptus* (*E. globulus* and *E. urograndis*) exhibit antimicrobial activity against planktonic and biofilm cells. The results indicate the ability of EGEO to cause the microbial death of *S. mutans*, and for this it required only 15 min of contact and thus gave the best results [41]. Our work confirms that the essential oil affects the spectra of both planktonic and biofilm cells [42,43]. Significant biofilm inhibition (83 ± 3% and 84 ± 5%) of *E. globulus* extract (leaf extract) was also observed for *S. aureus* and *P. aeruginosa* [44]. Maria et al. [45] have tested various essential oils, including EGEO, in studies and the results confirm our findings that essential oil has antibiofilm activity against several microorganisms. Additionally, the biofilm change can be observed when EGEO was added to the chitosan biofilm; especially at higher concentrations, the microstructure of the biofilm changed considerably and caused a heterogeneous structure [46].

The insecticidal activity of EGEO has been tested in several studies. Significant insecticidal activity (36.0–93.0%) was demonstrated by the essential oil against *M. domestica* L. [47]. It also showed effective activity against *T. confusum*, especially extracts from young *Eucalyptus* leaves. The highest mortality rate (31.67%) was obtained at a short time (2 h) [48]. The insecticidal activity of EGEO was tested at different concentrations of 0.5%, 0.3%, 0.2%, and 0.1% against the larval maturation and adult emergence of the housefly, *Musca domestica*. There was a larval mortality of 90%. On the other hand, developed pupae did not pupate adults [49]. *Eucalyptus globulus* was analyzed via gas chromatography/mass spectrometry (GC/MS) and their potential insecticidal activity against the olive fruit fly *Bactrocera oleae* was investigated [50]. The findings of the present study showed that EGEO against *B. oleae* is considered as an eco-friendly alternative natural insecticide [51,52,53].

## 4. Materials and Methods

### 4.1. Essential Oil

*Eucalyptus globulus* essential oil (EGEO) was obtained from Hanus s.r.o. (Nitra, Slovakia) which was cultivated in Spain, and it was prepared via steam distillation of fresh leaves. It is characterized by a strong and distinctive cineole aroma. It was stored in the dark at 4 °C.

### 4.2. Microorganisms

The microorganisms used in this work were purchased from a Czech collection of microorganisms (Brno, Czech Republic). Antimicrobial activity was investigated against the growth inhibition of 3 Gram-positive (G^+^) bacteria (*Staphylococcus aureus* subsp. *aureus* CCM 8223, *Bacillus subtilis* CCM 1999, *Enterococcus faecalis* CCM 4224,) and 3 Gram-negative (G^−^) bacteria (*Yersinia enterocolitica* CCM 7204, *Pseudomonas aeruginosa* CCM 3955, *Salmonella enterica* subsp. *enterica* CCM 4420) as well as the inhibitory effect against yeasts (*Candida albicans* CCM 8261, *Candida krusei* CCM 8271, *Candida glabrata* CCM 8270, *Candida tropicalis* CCM 8223). The effect of essential oil in the vapor phase was also tested against 3 microscopic fungi isolated from grapes (*Botrytis cinerea*, *Penicillium citrinum*, *Aspergilus flavus*). The biofilm-producing bacteria, *P. fluorescens*, which was isolated from fish, was used in the determination of antibiofilm activity. MALDI-TOF MS Biotyper and 16S rRNA sequencing identified biofilm-producing bacteria and microscopic filamentous fungi.

### 4.3. Chemical Characterization of Eucalyptus Globulus Essential Oil via Gas Chromatography/Mass Spectrometry (GC/MS) and Gas Chromatography (GC-FID)

The chromatographic analysis of volatile constituents presented in EGEO was carried out using a gas chromatograph (Agilent Technologies (Palo Alto, Santa Clara, CA, USA) 6890 N) equipped with a quadrupole mass spectrometer 5975 B (Agilent Technologies, Santa Clara, CA, USA), operated with an interfaced HP Enhanced ChemStation software (Agilent Technologies, Santa Clara, CA, USA). Employing the HP-5MS capillary column (30 m × 0.25 mm × 0.25 µm), the separation of volatiles was performed. Carrier gas helium 5.0 was used with a flow rate of 1 mL/min. The sample of essential oil was diluted with hexane (10% solution), while the injection volume was 1 µL. The temperature of the split/splitless injector, the MS source, and the MS quadruple were set at 280 °C, 230 °C, and 150 °C, respectively. The solvent delay time was 3 min, while the mass scan range was 35–550 amu at 70 eV. The conditions of the analysis (GC and GC–MS) were the following: a temperature program of 60 °C to 150 °C (rate of increase 3 °C/min), and 150 °C to 280 °C (rate of increase 5 °C/min), held for 4 min at 280 °C. The total run time was 60 min, and the split ratio was 40:8:1.

Constituents of the essential oil sample examined in this study were identified via a comparison of their retention indices (RI) as well as the reference spectra reported in the literature and the ones stored in the MS library (Wiley7Nist) [54,55]. Using GC-FID with the same HP-5MS capillary column performed was a semiquantification of the components taking into consideration amounts higher than 0.1%.

### 4.4. Determination of Antioxidant Activity Using ABTS Assay

For the purpose of determining the antioxidant capacity of EGEO, we performed the ABTS assay. The ABTS radical cation was generated according to the previously described method [18]. Briefly, via oxidation of the ABTS solution (7 mM) with 2.45 mM of the potassium persulfate solution, the ABTS radical cation stock solution was prepared and stored in the dark at 25 °C for 24 h. Prior to the analysis, the stock solution was diluted in methanol up to an absorbance value of 0.7 at 744 nm. In a 96-well microtiter plate, 190 μL of this solution was mixed with 10 μL of EO or Trolox (Sigma Aldrich, Schnelldorf, Germany) for 30 min with continuous shaking at 1000 rpm at room temperature in the dark. A decrease in the absorbance at 744 nm was registered using a microplate reader, and the results are presented as a percentage of the ABTS^•+^ inhibition and according to the calibration curve of Trolox (TEAC). As a standard reference, Trolox (1–5 mg/L in methanol) was used, while methanol (Uvasol^®^ for spectroscopy, Merck, Darmstadt, Germany) served as a blank. The percentage of the inhibition of ABTS^•+^ was calculated according to the following formula: (A0 − AA)/A0 × 100; where A0 is the absorbance of ABTS^•+^ and AA is the absorbance of the sample. All measurements were performed in triplicate, and the results were presented as the mean values ± standard deviation (SD) of three independent measurements.

### 4.5. Determination of Antimicrobial Activity via Disc Diffusion Method

We tested the antimicrobial activity of EGEO via the disc diffusion method. The assay was performed on Petri dish containing Mueller Hinton agar (MHA, Oxoid, Basingstoke, UK). A 100 μL of microorganism was added to the agar. The microorganism was incubated in a suitable culture medium for 24 h, with the bacteria in the Mueller Hinton broth (MHB, Oxoid, Basingstoke, UK) at 37 °C and the yeast and microscopic fungi in Sabouraud’s dextrose broth (SDB, Oxoid, Basingstoke, UK) at 25 °C, and then the optical density was adjusted using a densitometer to 0.5 McFarland standard (1.5 × 10^8^ CFU/mL). Place sterile blank discs (6 mm) on the prepared and inoculated Petri dishes and pipette 10 μL EGEO onto them. The antifungal activity of EGEO was analyzed against *P. citrinum*, *B. cinerea*, and *A. flavus*. Selected concentrations of essential oil (500, 250, 125, and 62.5 μL/mL) were prepared via dilution in 0.1% DMSO (Sigma Aldrich, Taufkirchen, Germany) solution. Petri dishes are cultured for 24 h at the appropriate temperature, bacteria at 37 °C, microscopic filamentous fungi at 25 °C, and then the zone of inhibition (radius) is measured in triplicate. A clear blank disc, without essential oil, was used as a negative control and an antibiotic disc (cefoxitin (G^−^), gentamicin (G^+^); Oxoid, Basingstoke, UK) and antifungal (fluconazole; Oxoid, Basingstoke, UK) was used as a positive control.

Inhibition zones were measured at three sides from the edge of the filter. An inhibition zone larger than 10 mm was determined as very strong antimicrobial activity, an inhibition zone of 10–5 mm was determined as moderate activity, and an inhibition zone of 5–1 mm was determined as weak activity. The antibiotics were used as a control, meropenem (Oxoid, Basingstoke, UK) for bacteria, and pure DMSO and fluconazole (Oxoid, Basingstoke, UK) for microscopic filamentous fungi. The method for evaluation of the inhibition zones was the same also for the biofilm-forming bacteria. Antimicrobial activity was measured in triplicate.

### 4.6. Minimal Inhibitory Concentration (MIC)

We tested EGEO to see what concentration could inhibit the growth of 50% and 90% of microorganisms using the minimal inhibitory concentration method (MIC). The assay was performed on a 96−well plate to which 100 μL of microorganisms at a density of 0.5 McFarland in Mueller Hinton Broth (MHB, Oxoid, Basingstoke, UK) were added and incubated for 24 h the day before at 37 °C for the bacteria and 25 °C for the yeast. An amount of 100 μL of EGEO was pipetted into the first column and the concentration was gradually diluted from 500 μL/mL to 0.244 μL/mL. The absorbance of the 96-well plate thus prepared was measured using a Glomax spectrophotometer (Promega Inc., Madison, WI, USA) at 570 nm, and the absorbance was measured again after a 24 h incubation. Pure MHB without the addition of essential oil and the microorganism was used as a negative control, and only the microorganism was used as a positive control (0.5 McFarland).

### 4.7. Analysis of Differences in Biofilm Development with MALDI-TOF MS Biotyper

The antibiofilm activity of EGEO, the ability to inhibit and degrade proteins of biofilm-forming bacteria *P. flourescens* biofilm, was analyzed via MALDI-TOF MS Biotyper mass spectrometry. A liquid culture medium, 20 mL Mueller Hinton broth (MHB, Oxoid, Basingstoke, UK), was added to the polypropylene centrifuge tubes containing different test surfaces of 50 mL (stainless steel and plastic), with a length of about 5 cm and a thickness of about 1 cm being placed inside the tubes. EGEO (0.1% (*w*/*v*)) was added to the tubes with the culture medium in addition to the biofilm-forming bacteria. A 100 µL of bacterial inoculum with an optical density of 0.5 McF (1.5 × 10^8^ CFU/mL) was added to the tubes. The tubes thus prepared were incubated on a shaker (GFL 3031, Germany) during constant agitation at 37 °C and 170 rpm. The tubes were sampled during days 3, 5, 7, 9, 12, and 14 of the experiment and the effect of EGEO on biofilm formation was detected as a function of time using MALDI-TOF mass spectrometry. Using cotton swabs, the sample was collected from the surface of the materials present in the tubes and specified on a MALDI-TOF metal target plate. Samples were also taken from free planktonic cells that were present in the medium. An amount of 300 µL of medium where planktonic cells were present was collected and centrifuged at 12.000 rpm for 1 min and the pellet was rinsed 3 times and washed in 30 µL of ultrapure water. The purified pellet was then dissolved in 30 µL of ultrapure water and 1 µL of it was loaded onto a MALDI-TOF plate. The target metal plate was left at room temperature until the samples were dry. When the samples were dry, 1 µL of α-cyano-4-hydroxycinnamic acid matrix (10 mg/mL) was added. After the matrix had dried and then crystallized uniformly, the samples were analyzed with a MALDI-TOF MicroFlex (Bruker Daltonics) using the linear, positive mode setting, where the m/z range was 200–2000. Automated analyses were used to generate standard global spectra (MSP). MALDI Biotyper 3.0 generated 19 spectra from which dendrograms were merged and prepared [56].

### 4.8. Antimicrobial Analysis In Situ (Vapor Phase) on a Food Model

EGEO was also tested in this work for its antifungal effect in the vapor phase and inhibitory effect on the growth of microscopic fungi *P. citrinum*, *B. cinerea*, and *A. flavus*. The antibacterial effect in the vapor phase against the *Salmonella enterica*, *Y. enterocolitica*, *B. subtilis*, *E. faecalis*, *S. aureus*, *P. aeroginosa*, and *P. flourescens* biofilms was also tested. The inhibitory effect of EGEO on yeast growth was tested on 4 yeasts: *C. albicans*, *C. glabrata*, *C. krusei*, and *C. tropicalis*. MHA (Oxoid, Basingstoke, UK) was added to the Petri dishes, both to the bottom of the dish and to the lid, for sufficient closure. White radish was used as a food model, which was rinsed 3 times in distilled water after being cut into rounds (0.5 mm). The white radish was inoculated with the selected microorganism and bacteria were incubated on Tryptone Soya Agar (TSA, Oxoid, Basingstoke, UK) for 24 h at 37 °C, yeasts and microscopic fungi on Sabouraud Dextrose Agar (SDA, Oxoid, Basingstoke, UK) for 5 days at 25 °C, in a single injection using disposable bacterial eyelets. Filter paper cut to the size of the lid (6 cm) of a Petri dish was placed on the lid and then pipetted with 100 μL of selected concentrations (62.5, 125, 250, and 500 µL/L diluted in ethyl acetate) of EGEO. The control group remained untreated. Petri dishes were hermetically sealed and incubated in the dark for 14 days at 25 °C for the fungi and yeast and for bacteria it was incubated for 7 days at 37 °C. Measurements were performed in triplicate.

The growth inhibition of microorganisms was evaluated via the stereological method. The volumetric density (Vv) of mycobacteria was determined using ImageJ software. Subsequently, the colony (P) and substrate (p) stereological network points were counted. The growth density of the microorganisms was calculated as a perentage using the formula Vv = P/p × 100. The antibacterial, antifungal, and antifungal activity of the essential oil in the vapor phase was expressed as the growth inhibition of mycobacteria in a percentage (MGI): MGI = [(C − T)/C] × 100, where C was the growth density of mycobacteria in the control group and T was the growth density of mycobacteria in the treated group [57,58].

### 4.9. Insecticidal Activity of Eucalyptus globulus Essential Oil

The effect of EGEO on insecticidal activity against *Oxycarenus lavaterae* was tested. Thirty individuals were placed in Petri dishes with vents and divided into multiple Petri dishes. A circle-shaped filter paper (6 cm) was inserted into the lid of the Petri dish where selected concentrations (50%, 25%, 12.5%, 6.25%, and 3.125%) of essential oil were added and diluted with 0.1% polysorbate solution in a volume of 100 µL. The Petri dish thus prepared containing 30 individuals of *Oxycarenus lavaterae* and the appropriate concentration of essential oil was sealed using parafilm. After 24 h and at laboratory temperature, the effect of EGEO on the viability of *Oxycarenus lavaterae* was evaluated. A 0.1% solution of polysorbate was used as a control. Insecticidal activity was calculated from the ratio of the number of dead to the number of live individuals. The experiment was carried out in three repetitions.

### 4.10. Statistical Data Evaluation

The data were analyzed using one-way ANOVA with Prism 8.0.1 (GraphPad Software, San Diego, CA, USA), followed by Tukey’s test at a p-value of less than 0.05. MIC values, representing the concentration at which 50% and 90% inhibition of bacterial growth occurred, were determined through logit analysis. The data processing was performed using SAS^®^ version 8 software. All measurements and analyses were carried out in triplicate.

## 5. Conclusions

The main components of EGEO were 1,8-cineole (63.1%), *p*-cimene (7.7%), a-pinene (7.3%), and a-limonene (6.9%). Up to 99.2% of monoterpenes were present. The antioxidant potential of essential oil and the results indicate that 10 μL of this sample can neutralize 55.44 ± 0.99% of ABTS^•+^, which is equivalent to 3.22 ± 0.01 TEAC. We rate this antioxidant activity as high. Antimicrobial activity determined via the disc diffusion method was confirmed to have the strongest activity against yeasts and microscopic fungi (11.00 ± 0.00–14.00 ± 1.00 mm). The minimum inhibitory concentration results show that the lowest concentration (MIC 50 2.93 µL/mL; MIC 90 3.17 µL/mL) and the strongest antimicrobial activity were also recorded against the yeast *C. tropicalis*. The minimum inhibitory concentration against microscopic fungi shows the most effective EGEO concentration of 500 µL/mL. For in situ antimicrobial activity, EGEO in the vapor phase was found to be most effective against G^−^ at a concentration of 500 µL/mL. Antibiofilm activity was also confirmed as we detected structural changes in *P. fluorescens* biofilm using MALDI-TOF MS Biotyper. Concentrations of 100, 50, and 25% of EGEO showed 100% insecticidal activity against *O. lavaterae*. EGEO was comprehensively investigated in this study and information regarding the biological activities and chemical composition of the essential oil of *Eucalyptus globulus* was expanded.

## Figures and Tables

**Table 1:** Chemical Composition of *Eucalyptus globulus* Essential Oil.

No	RI ^a^	Compound ^b^	%
1	909	isobutyl isobutyrate	0.1
2	926	a-thujene	0.4
3	938	a-pinene	7.3
4	948	camphene	0.8
5	977	sabinene	1.0
6	980	b-pinene	3.0
7	992	b-myrcene	1.7
8	1004	a-phellandrene	1.0
9	1009	d-3-carene	0.1
10	1016	a-terpinene	1.0
11	1023	*p*-cimene	7.7
12	1028	a-limonene	6.9
13	1033	1,8-cineole	63.1
14	1047	(*E*)-b-ocimene	0.2
15	1060	g-terpinene	3.6
16	1088	a-terpinolene	0.6
17	1140	trans-pinocarveol	0.1
18	1148	camphor	0.1
19	1151	menthone	0.2
20	1160	pinocarvone	0.1
21	1178	4-terpinenol	0.2
22	1189	a-terpineol	0.1
26	1443	aromadendrene	0.4
27	1498	ledene	tr ^c^
	Total		99.7

^a^ Values of retention indices on HP-5MS column; ^b^ identified compounds; ^c^ tr—compounds identified in amounts less than 0.1%.

**Table 2:** The volatiles presented in percentage for each class of compounds.

Class of Compounds	%
monoterpenes	99.2
monoterpene hydrocarbons	35.3
oxygenated monoterpenes	63.9
monoterpene epoxide	63.1
monoterpene alcohols	0.4
monoterpene ketones	0.4
sesquiterpenes	0.4
sesquiterpene hydrocarbons	0.4
oxygenated sesquiterpenes	Tr
sesquiterpene alcohols	Tr
non-terpenic	0.1
ester	0.1
Total	99.7

**Table 3:** Antimicrobial activity of *Eucalyptus globulus* Essential Oil.

Microorganism	Inhibition Zone (mm)	Activity of EO	Control
Gram-positive bacteria			
*Bacillus subtilis*	6.67 ± 0.58	**	33 ± 1.00
*Enterococcus faecalis*	2.33 ± 0.58	*	29 ± 0.50
*Staphylococcus aureus*	5.67 ± 0.58	**	32 ± 1.00
Gram-negative bacteria			
*Pseudomonas aeruginosa*	4.33 ± 0.58	*	25 ± 1.00
*Salmonella enterica*	5.10 ± 1.00	**	27 ± 2.00
*Yersinia enterocolitica*	5.33 ± 0.58	**	27 ± 1.50
*Pseudomonas fluorescens* biofilm	3.67 ± 0.58	*	28 ± 1.00
Yeasts			
*Candida albicans*	14.00 ± 1.00	***	28 ± 2.00
*Candida glabrata*	7.33 ± 0.58	**	33 ± 1.50
*Candida krusei*	4.33 ± 0.58	*	33 ± 3.00
*Candida tropicalis*	5.05 ± 1.00	**	33 ± 1.00
Fungi			
*Aspergillus flavus*	11.00 ± 0.00	***	32 ± 0.58
*Botrytis cinerae*	11.67 ± 0.58	***	33 ± 1.00
*Penicillium citrinum*	12.33 ± 0.58	***	31 ± 0.58

* Weak activity (zone 1–5 mm); ** moderate activity (zone 5–10 mm); *** strong activity (over 10 mm); antibiotics used as control: cefoxitin for G^−^ bacteria, gentamicin for G^+^ bacteria, fluconazole for microscopic filamentous fungi.

**Table 4:** Minimal inhibition concentration of *Eucalyptus globulus* Essential Oil.

Microorganism	MIC 50 (µL/mL)	MIC 90 (µL/mL)
Gram-positive bacteria		
*Bacillus subtilis*	374.02	397.64
*Enterococcus faecalis*	6.37	22.44
*Staphylococcus aureus*	140.25	334.72
Gram-negative bacteria		
*Pseudomonas aeruginosa*	374.02	397.64
*Salmonella enterica*	15.62	49.67
*Yersinia enterocolitica*	46.89	50.07
*Pseudomonas fluorescens* biofilm	93.80	99.91
Yeasts		
*Candida albicans*	77.21	86.42
*Candida glabrata*	245.02	295.79
*Candida krusei*	5.86	6.31
*Candida tropicalis*	2.93	3.17

**Table 5:** Minimal inhibition concentration of microscopic fungi.

Fungi	Inhibition Zone (mm)
*Aspergillus flavus*	
500 µL/mL	7.67 ± 0.58
250 µL/mL	5.00 ± 1.00
125 µL/mL	5.00 ± 0.58
62.5 µL/mL	3.33 ± 1.53
*Botrytis cinerea*	
500 µL/mL	6.33 ± 0.58
250 µL/mL	4.33 ± 2.06
125 µL/mL	7.33 ± 2.89
62.5 µL/mL	5.00 ± 2.65
*Penicillium citrinum*	
500 µL/mL	4.33 ± 1.53
250 µL/mL	0.00 ± 0.00
125 µL/mL	0.00 ± 0.00
62.5 µL/mL	0.00 ± 0.00

**Table 6:** In situ analysis of the antimicrobial activity of the vapor phase of *Eucalyptus globulus* essential oil on white radish.

	White Radish			
Bacterial Growth Inhibition (%)	Gram-Positive Bacteria			
*Eucalyptus globulus* EO (µL/L)	*B. subtilis*	*E. faecalis*	*S. aureus*	
62.5	−43.94 ± 1.98 ^a^	76.86 ± 1.43 ^d^	76.79 ± 2.15 ^d^	
125	−24.74 ± 2.05 ^b^	54.15 ± 2.47 ^c^	5.67 ± 1.03 ^a^	
250	24.92 ± 1.68 ^d^	33.41 ± 1.48 ^b^	13.49 ± 2.59 ^b^	
500	−11.92 ± 0.66 ^c^	13.59 ± 2.80 ^a^	56.97 ± 2.22 ^c^	
Bacterial Growth Inhibition (%)	Gram-negative bacteria			
*Eucalyptus globulus* EO (µL/L)	*P. flourescens* biofilm	*P. aeroginosa*	*S. enterica*	*Y. enterocolitica*
62.5	43.69 ± 1.92 ^d^	8.62 ± 1.06 ^a^	13.33 ± 1.94 ^a^	15.44 ± 2.64 ^a^
125	35.00 ± 2.69 ^c^	24.74 ± 2.10 ^b^	75.29 ± 2.91 ^d^	65.75 ± 1.86 ^c^
250	−54.41 ± 2.13 ^a^	34.08 ± 1.99 ^c^	24.70 ± 2.82 ^b^	33.37 ± 1.47 ^b^
500	25.13 ± 2.04 ^b^	86.79 ± 2.40 ^d^	44.11 ± 1.43 ^c^	91.26 ± 4.58 ^d^
Mycelial Growth Inhibition (%)	Yeasts			
*Eucalyptus globulus* EO (µL/L)	*C. albicans*	*C. glabrata*	*C. krusei*	*C. tropicalis*
62.5	5.97 ± 1.36 ^a^	86.82 ± 3.07 ^d^	11.71 ± 1.00 ^a^	35.57 ± 2.04 ^c^
125	64.97 ± 2.56 ^d^	−8.00 ± 1.60 ^a^	34.07 ± 2.53 ^b^	8.26 ± 1.35 ^b^
250	13.34 ± 2.19 ^b^	65.96 ± 1.71 ^c^	66.26 ± 2.35 ^c^	55.97 ± 2.97 ^d^
500	54.29 ± 2.50 ^c^	14.69 ± 2.77 ^b^	75.97 ± 2.98 ^d^	−24.37 ± 2.90 ^a^
Mycelial Growth Inhibition (%)	Microscopic fungi			
*Eucalyptus globulus* EO (µL/L)	*A. flavus*	*B. cinerea*	*P. citrinum*	
62.5	76.67 ± 2.77 ^d^	76.06 ± 2.77 ^d^	85.77 ± 1.71 ^d^	
125	55.00 ± 2.62 ^c^	56.01 ± 2.34 ^c^	63.74 ± 2.06 ^c^	
250	43.37 ± 2.06 ^b^	34.67 ± 2.90 ^b^	33.10 ± 1.44 ^a^	
500	14.68 ± 2.05 ^a^	11.70 ± 0.87 ^a^	54.30 ± 2.24 ^b^	

One-way ANOVA; individual letters (^a–d^) in upper case indicate the statistical differences between the concentrations, *p* ≤ 0.05; the negative values indicate the probacterial activity of the essential oil against the growth of microbial strains.

**Table 7:** Insecticidal activity of *Eucalyptus globulus* essential oil.

Concentration (%)	Number of Living Individuals	Number of Dead Individuals	Insecticidal Activity (%)
100	0	30	100.00
50	0	30	100.00
25	0	30	100.00
12.5	12	18	60.00
6.25	24	6	20.00
3.125	28	2	6.66
Control group	30	0	0.00

## Data Availability

All data generated or analyzed during this study are included in this published article.
